# Associations between cancer history, social distancing behaviors, and loneliness in adults during the COVID-19 pandemic

**DOI:** 10.1371/journal.pone.0281713

**Published:** 2023-02-16

**Authors:** Jenny Yang, Xiaochen Zhang, Mengda Yu, James L. Fisher, Electra D. Paskett

**Affiliations:** 1 Medical Student Research Program, College of Medicine, The Ohio State University, Columbus, OH, United States of America; 2 Comprehensive Cancer Center, The Ohio State University, Columbus, OH, United States of America; 3 Center for Biostatistics, The Ohio State University, Columbus, OH, United States of America; 4 James Cancer Hospital and Solove Research Institute, Columbus, OH, United States of America; 5 College of Public Health, The Ohio State University, Columbus, OH, United States of America; 6 College of Medicine, The Ohio State University, Columbus, OH, United States of America; UCSD: University of California San Diego, UNITED STATES

## Abstract

**Background:**

During the COVID-19 pandemic, enforced social distancing initiatives have highlighted differences in social distancing practices and the resulting loneliness in various populations. The objective of this study was to examine how cancer history and social distancing practices relate to loneliness during COVID-19.

**Methods and findings:**

Participants from previous studies (N = 32,989) with permission to be re-contacted were invited to complete a survey online, by phone, or by mail between June and November 2020. Linear and logistic regression models were used to determine the associations between cancer history, social distancing, and loneliness.

**Results:**

Among the included participants (n = 5729), the average age was 56.7 years, 35.6% were male, 89.4% were White, and 54.9% had a cancer history (n = 3147). Individuals with a cancer history were more likely to not contact people outside of their household (49.0% vs. 41.9%, p<0.01), but were less likely to feel lonely (35.8% vs. 45.3%, p<0.0001) compared to those without a cancer history. Higher adherence to social distancing behaviors was associated with higher odds of loneliness among individuals with (OR = 1.27, 95% CI: 1.17-1.38) and without a cancer history (OR = 1.15, 95% CI: 1.06-1.25).

**Conclusions:**

Findings from this study can inform efforts to support the mental health of individuals susceptible to loneliness during the COVID-19 pandemic.

## Introduction

The COVID-19 pandemic has caused an unprecedented impact on the entire world, affecting people of all backgrounds. To mitigate the spread of the virus, widespread social distancing initiatives were enacted, impacting the everyday lives of individuals [1]. The abilities and willingness to prioritize practicing social distancing have highlighted inequalities between different populations [2]. Restrictions due to lockdown measures and stay-at-home orders can also have various consequences on one’s health, such as feelings of loneliness [3, 4].

While most people were encouraged to practice social distancing to minimize the spread of COVID-19, the degree of adherence varied. Individuals with underlying diseases, such as cancer survivors, were discovered to adhere well to preventive measures, including social distancing, wearing a face mask, and avoiding crowded areas [2, 5]. This could be due to the Centers for Disease Control and Prevention’s (CDC) emphasis on the importance of social distancing for those with weakened immune systems, such as patients with cancer treated with chemotherapy [6]. Another factor that influences social distancing practices is county of residence. For example, rural residents were found to be less adherent to preventive policies during the pandemic, and governors of states with large rural populations were also slower to adopt social distancing policies [2, 7].

The degree to which an individual practices preventive behaviors such as social distancing could be associated with feelings of loneliness. Previous research has discovered that more individuals with chronic conditions reported feeling lonely following one month of social distancing compared to before the pandemic [8]. Additionally, enforced social distancing was the strongest predictor of loneliness among patients with cancer [9]. Individuals with limited access to online resources to maintain social relationships, such as rural residents with less access to the internet than metro residents, may also be susceptive to feeling lonely during the pandemic [10, 11]. Prior research has found that feelings of loneliness and lack of social support may be associated with a negative impact on cancer-specific mortality, severity of symptoms, and depression among cancer survivors [12–14]. Therefore, loneliness that individuals may experience while practicing social distancing during the pandemic may have similar negative consequences.

Due to the novelty of the COVID-19 pandemic, there remain several gaps in knowledge on social distancing and loneliness. First, research comparing differences in social distancing practices between individuals with and without a history of cancer is sparse. Additionally, studies examining the relationship between social distancing and feelings of loneliness often focus on one specific group and lack diversity. Therefore, this study examined: 1) how cancer history correlated with the degree to which individuals practiced social distancing and how often they felt lonely in the seven days prior to taking the survey, 2) how social distancing practices during the COVID-19 pandemic relate to feelings of loneliness, and 3) how the interaction between cancer history and metro residency changed the associations between cancer history, social distancing practices, and feelings of loneliness. Findings from this study can support efforts to mitigate feelings of loneliness in individuals struggling during the pandemic as well as inform future research examining how loneliness is associated with adverse health outcomes and affects overall well-being.

## Materials and methods

### Overview

This study was part of an NCI-funded initiative conducted in conjunction with 16 other NCI-designated Cancer Centers, the IC-4 (Impact of COVID-19 on the Cancer Continuum Consortium). The initiative was funded to work collectively to develop core survey items and implement population surveys in the respective catchment areas. The overall goal of the IC-4 was to assess how differences in demographics (rural/urban, age, gender, race, educational attainment) impact engagement in cancer preventive behaviors (e.g., tobacco cessation, screening, diet) and cancer management/survivorship behaviors (e.g., adherence to treatment, adherence to surveillance, access to health services) in the context of COVID-19 environmental constraints (e.g., social distancing, employment, mental health). Each site had its own theoretical framework and survey methods. Our site used the IC-4 core set of common data elements, with remote data collection methods to include many unique and diverse populations. This study was approved by the OSU Institutional Review Board in June 2020.

### Theoretical framework

This study was grounded in the Health Belief Model (HBM) [15, 16]. According to the HBM, individuals’ change in health behaviors depends on a series of health beliefs, including: 1) perceived susceptibility to COVID-19 exposure, 2) perceived severity of the consequences of contracting COVID-19 (e.g., hospitalization or death), 3) perceived benefits of the effectiveness of the proposed COVID-19 prevention measures, 4) perceived barriers to executing the proposed prevention measures, 5) cue to the proposed prevention actions and 6) self-efficacy in the person’s ability to successfully perform COVID-19 prevention measures.

### Survey development

The survey elements were finalized in conjunction with other members of the IC-4 [17, 18]. The survey included individual behaviors related to the mitigation of COVID-19 transmission, the challenges related to social distancing, and self/family isolation, stress, and health behaviors that are highly relevant to cancer and other chronic diseases. Questions also assessed perceived stigma related to COVID-19 with respect to different population groups and covariates, such as health literacy and mental health, suspected of moderating these influences.

### Sample selection

Participants who agreed to be re-contacted from previous studies of the study authors were asked to participate in this study, with a wide variety of populations including healthy residents and cancer patients. Eligible participants were adults aged 18 years or older who consented to take part in the study. To ensure the inclusion of the most vulnerable, underserved, and minority populations, we sought to recruit healthy adult volunteers, cancer patients, cancer survivors, and cancer patients’ and survivors’ caregivers, mostly from Ohio, with some from Indiana and other states. This was achieved by employing two recruitment strategies. First, we identified and contacted individuals who previously participated in studies conducted at OSU and consented to be contacted for future research projects. In addition, we invited cancer patients and survivors to nominate their primary caregivers to participate in the study. The list of previous research projects conducted at OSU included the Rural Interventions for Screening Effectiveness (RISE) study (R01 CA196243), the Community Initiative Towards Improving Equity and Health Status (CITIES) cohort (Supplement to P30CA016058), the Buckeye Teen Health Study (BTHS) study (P50CA180908), the Ohio State University Center of Excellence in Regulatory Tobacco Science (CERTS) cohort (P50CA180908), and members of the Total Cancer Care (TCC) cohort (P30CA016058). Second, to further enhance the representativeness of our study sample and ensure the inclusion of minority and underserved communities, we utilized our community partners and listservs to send tailored email invitations.

### Interview/data collection

We utilized several data collection methods, including web, phone, and mailed surveys. Respondents with valid emails received an initial survey invitation email along with three reminders seven days apart. All participants were initially screened using an eligibility form before conducting the survey. Participants were able to save their responses in the web survey and resume it at a later time. Those who partially completed the web survey received an email reminder one week after they last accessed the survey. A trained interviewer contacted participants without an email address and those with invalid emails on file by phone. Participants who were initially reached by phone were offered the option to complete the survey over the phone or online. We mailed a cover letter and a paper survey with a self-addressed, stamped return envelope to participants who requested a mailed survey. For Non-English-speaking participants, a bilingual staff member administered the survey in the appropriate language. Participants were offered a $10 gift card upon completion of the survey. All data were collected and managed using the Research Electronic Data Capture (REDCap) secure web-based application hosted at OSU.

### Measurements

#### Social distancing measures

Social distancing behaviors were measured by 5 items: 1) “Staying at home except for going to work, outdoors to exercise, or going to the grocery store, pharmacy, or to get medical care”; 2) “Not having relatives, friends, or neighbors come into your home?”; 3) “Staying 6 feet away from people when you leave your home?”; 4) “Wearing a face covering when you are outdoors?”; and 5) “Wearing a face covering when you are inside a store or other place besides your home?” Answering “yes” to the above questions was defined as participation in that social distancing behavior and coded as 1. Answering “no” was coded as 0. The scores for each item were summed and ranged from 0–5, with higher scores reflecting participation in more social distancing behaviors.

Participation in social gatherings was assessed through 2 questions. They included did you attend: 1) “Any gatherings, not including work, with more than 2 people who do not live in the same house as you?” and 2) “Other large social gatherings of 20 or more people?” Responses consisted of “yes” (1) or “no” (0). Scores for each item were summed up to a score ranging from 0–2, with higher scores representing more attendance to social gatherings.

Social contact was determined by the question “In the past 2 weeks, with how many people outside of your household have you been in close contact (within 6 feet) for 4 hours or more in a single day?” Participants provided a numerical value, which were categorized into 3 levels: 0, 1–3, or ≥4.

#### Loneliness

The outcome measure for this study was loneliness. This was defined by responses to the following: “In the past 7 days, how often have you felt lonely?” Responses were dichotomized into 2 levels: 1) “Not at all or less than 1 day” and 2) “1 day or more.”

In terms of history of cancer diagnosis, participants were asked whether a doctor had ever diagnosed them with cancer.

Demographic variables collected included age, sex assigned at birth (male, female), race (White, Black/African American, Asian, Multiple/Other), marital status (Single or never married, Married/Living as Married, Divorced/Widowed/Separated/Other), possession of a device for video conferencing, and total number of people living in the household (adults and children). The county of residence of participants was coded to metro and non-metro based on the 2013 Rural-Urban Continuum Codes (RUCC) [19]. Counties with RUCC 1 to 3 were coded as metro while those with RUCC 4 to 9 were coded as non-metro.

Socioeconomic status (SES) was defined using a modified Hollingshead Score from 4 items [20]: education, income, occupation, and insurance status. Participants were asked the highest grade or level of school and were classified to “High School (HS) or less,” “Some college/Associate Degree,” “BS/BA,” and “MS/MA or more.” For income, participants were asked “Thinking about members of your family living in your household, what is the combined annual income, meaning the total pre-tax income from all sources earned in the past year?” Answers were divided into “<$35K,” “$35K to $49,999,” “$50K to $74,999,” and “$75K+.” The responses for education and income were coded as 0, 1, 2, and 3 respectively. For employment, participants were categorized into “Other,” “Retired,” and “Employed full or part time” coded as 0, 1, and 2 respectively. Lastly, individuals were asked to specify their insurance, which was sorted into “No insurance,” “Public only,” “Private only,” and “Public and Private.” For these responses, the first two were coded as 0 and 1, respectively, while the last two were both coded as 2. The scores for each of the four items were summed up to a score with a possible range of 0–10. Scores were then categorized into 3 levels based on quartiles, with higher scores indicating a higher SES.

### Data analysis

Characteristics were summarized using descriptive statistics, including means and standard deviations for the continuous variables and frequencies for the categorical variables overall, and by cancer history. Differences between those who had cancer diagnosis vs. no history of cancer diagnosis were compared using the two-sample T-test or Kruskal-Wallis test for the continuous variables and the Chi-square test or Fisher’s exact test for the categorical variables.

Linear regressions were used to assess the association between metro residency, social distancing behaviors, and social gathering behaviors by cancer history. Logistic regression was used to assess the association between metro residency and contacting any people outside of the household (yes/no) by cancer history. To examine whether the association of social distancing measures and metro residency differed by cancer history, an interaction term “cancer*metro” was included and tested using the adjusted Wald test. To assess how social distancing behaviors, social gathering behaviors, and the number of people contacted outside of the household were associated with loneliness, logistic regression was used by cancer history. To examine whether the association of loneliness and metro residency differs by cancer history, the interaction term “cancer*metro” was included in the full model. All multivariate models were adjusted for age, sex, race, SES group, marital status, number of people in the household, and the possession of a device for video conferencing. All analyses were conducted using SAS v9.4, with a significance level of 0.05.

## Results

### Sample description

**Fig 1:**
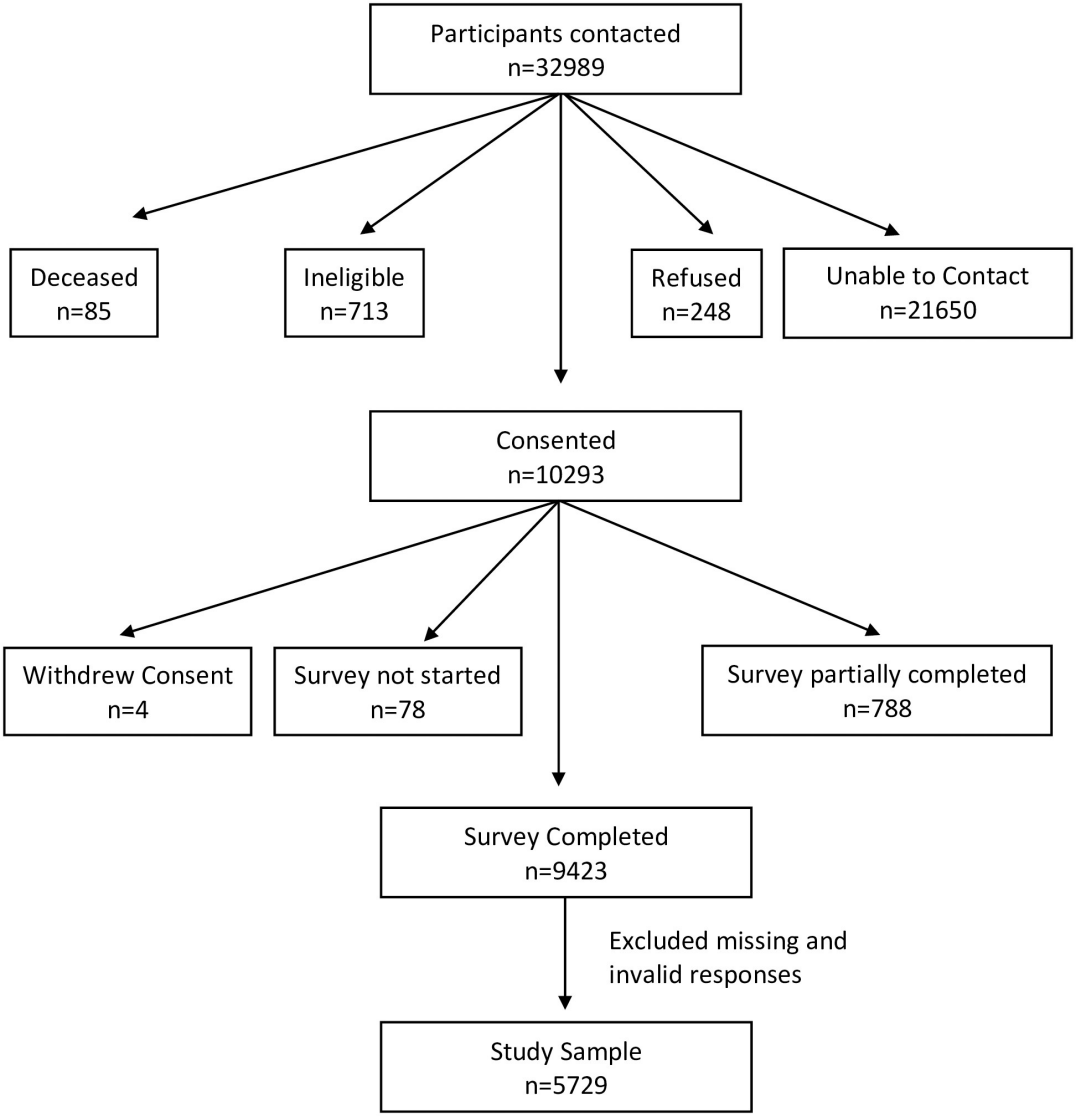
COVID-19 survey recruitment diagram.

A total of 32989 participants from previous studies of the study authors who agreed to be re-contacted were invited to partake in this study. Participants consisted of healthy individuals and cancer patients aged 18 or older mostly from Ohio, with some from Indiana and other states. Out of all contacted individuals, 9423 completed the survey, resulting in a response rate of 28.6% (Fig 1). After removal of missing and invalid responses (n = 3694, 39.2%), this current analysis consisted of 5729 participants. Compared to individuals included in the analysis, individuals excluded from the analysis were more likely to be female, of a lower SES, not married, living in one- or two-person households, without a video conferencing device, a resident of a non-metro county, and without a history of cancer (S1 Table).

Among the included participants, 54.9% had a cancer history (n = 3147) (Table 1). The average age was 56.7 years. 35.6% were male, 89.4% were White, 74.7% were married, 74.5% lived in metro counties, and 95.4% had a video conferencing device. Additionally, 21.8% were of low SES, 41.1% were intermediate SES, and 37.0% were high SES. About half of the participants lived in two-person households, 20.7% lived in households of ≥4 people, 17.9% lived in 3-person households, and 15.9% lived alone.

**Table 1:** Demographic characteristics of participants stratified by cancer history.

Variable	Total (n = 5729)	Individuals without cancer history (n = 2582)	Individuals with cancer history (n = 3147)	p-value
**Age, mean±SD, yrs**	56.69±13.59	52.04±13.63	60.49±12.31	<.0001
≤40 yrs	838 (14.63%)	592 (22.93%)	246 (7.82%)	
41 − 60 yrs	2377 (41.49%)	1240 (48.02%)	1137 (36.13%)	<.0001
≥61 yrs	2514 (43.88%)	750 (29.05%)	1764 (56.05%)	
**Sex**				
Male	2039 (35.59%)	794 (30.75%)	1245 (39.56%)	<.0001
Female	3690 (64.41%)	1788 (69.25%)	1902 (60.44%)	
**Race**				
White	5119 (89.35%)	2235 (86.56%)	2884 (91.64%)	<.0001
Black/African American	335 (5.85%)	186 (7.2%)	149 (4.73%)	
Asian	112 (1.95%)	61 (2.36%)	51 (1.62%)	
Other/Multiple	163 (2.85%)	100 (3.87%)	63 (2%)	
**Marital Status**				
Single, never married	532 (9.29%)	293 (11.35%)	239 (7.59%)	<.0001
Married/Living as married	4282 (74.74%)	1890 (73.2%)	2392 (76.01%)	
Divorced/Widowed/Separated/Other	915 (15.97%)	399 (15.45%)	516 (16.4%)	
**Socioeconomic Status group**				
low (score: 0–5)	1252 (21.85%)	548 (21.22%)	704 (22.37%)	<.0001
intermediate (score: 6–8)	2355 (41.11%)	969 (37.53%)	1386 (44.04%)	
high (score: 9–10)	2122 (37.04%)	1065 (41.25%)	1057 (33.59%)	
**Total number of people in household**				
1	911 (15.9%)	355 (13.75%)	556 (17.67%)	<.0001
2	2609 (45.54%)	937 (36.29%)	1672 (53.13%)	
3	1026 (17.91%)	556 (21.53%)	470 (14.93%)	
≥ 4	1183 (20.65%)	734 (28.43%)	449 (14.27%)	
**Possess device for video conferencing**				
No	262 (4.57%)	140 (5.42%)	122 (3.88%)	0.005
Yes	5467 (95.43%)	2442 (94.58%)	3025 (96.12%)	
**County of Residence**				
Non-Metro	1463 (25.54%)	709 (27.46%)	754 (23.96%)	0.003
Metro	4266 (74.46%)	1873 (72.54%)	2393 (76.04%)	

Compared to those without a cancer history, participants with a cancer history were more likely to be older, male, White, married, of intermediate SES, living alone or in two-person households, residing in metro counties, and in possession of a video conferencing device (Table 1; all p<0.05).

### Social distancing measures

Most individuals participated in four or five social distancing behaviors (Table 2, 36.2% and 33.1%), including staying at home, not having people come over, staying 6 feet away from people, wearing a face-covering outdoors, and wearing a face-covering inside a store or other place. Social distancing behaviors did not differ by cancer history (p = 0.41). About half of participants reported practicing one of the two social gathering behaviors, 19.4% reported practicing the two social gathering behaviors, and 31.5% attended no gathering or large social events. Similarly, social gathering behaviors did not differ by cancer history (p = 0.63). In terms of people contacted outside of the household in the past two weeks, 45.8% reported no contact with people outside of the household, 25.6% reported contacting one to three people, and 28.6% reported contacting four or more people. Individuals with a cancer history were more likely to have no contact with anyone outside the household compared to individuals without cancer (49.0% vs. 41.9%, p<0.0001).

**Table 2:** Social distancing measures of adults during the COVID-19 pandemic stratified by cancer history.

Variable	Total (n = 5729)	Individuals without cancer history (n = 2582)	Individuals with cancer history (n = 3147)	p-value
**Social Distancing Behaviors** ^1^				
0	56 (0.98%)	31 (1.2%)	25 (0.79%)	0.411
1	154 (2.69%)	71 (2.75%)	83 (2.64%)	
2	462 (8.06%)	221 (8.56%)	241 (7.66%)	
3	1089 (19.01%)	486 (18.82%)	603 (19.16%)	
4	2073 (36.18%)	912 (35.32%)	1161 (36.89%)	
5	1895 (33.08%)	861 (33.35%)	1034 (32.86%)	
**Social Gathering Behaviors** ^2^				
0	1802 (31.45%)	828 (32.07%)	974 (30.95%)	0.632
1	2818 (49.19%)	1263 (48.92%)	1555 (49.41%)	
2	1109 (19.36%)	491 (19.02%)	618 (19.64%)	
**Number of people contacted outside of household in past 2 weeks**				
0	2626 (45.84%)	1083 (41.94%)	1543 (49.03%)	<.0001
1–3	1465 (25.57%)	701 (27.15%)	764 (24.28%)	
≥4	1638 (28.59%)	798 (30.91%)	840 (26.69%)	
**Felt lonely in the past 7 days**				
<1 day	3433 (59.92%)	1413 (54.73%)	2020 (64.19%)	<.0001
≥ 1 day	2296 (40.08%)	1169 (45.27%)	1127 (35.81%)	

^1^social distancing behaviors include: staying at home except for going to work, outdoors to exercise, or going to the grocery store, pharmacy, or to get medical care; Not having people come into your home; Staying 6 feet away from people; Wearing a face covering when outdoors; Wearing a face covering when inside a store or other place.

^2^social gathering behaviors include gathering with more than 2 people and large social gathering ≥20 people

### Loneliness

Less than half of the participants reported that they felt lonely one day or more in the past seven days. Individuals with a cancer history were less likely to report feeling lonely one day or more compared to individuals without a cancer history (35.8% vs. 45.3%, p<0.0001).

#### Associations between metro residency and social distancing measures by cancer history

When looking at the social distancing behaviors, among individuals without a cancer history, Black and Asian individuals and those who lived in metro counties were adherent to more social distancing behaviors including staying at home, staying six feet apart from others, and wearing a mask (Table 3). Participants with intermediate or high SES or those with larger households were adherent to fewer social distancing behaviors. However, among individuals with a cancer history, older age, being female, those who were Black, Asian, or other race, had a video conferencing device, and those who lived in metro counties were adherent to more social distancing behaviors. Individuals who were married or living as married, divorced, widowed, or separated adhered to fewer social distancing behaviors. There was a significant interaction between cancer history and metro residency (p interaction = 0.001): the difference between metro and rural residence in social distancing behaviors is smaller among individuals with a cancer history (Δ number of social distancing behaviors = 0.13, 95% CI: 0.04–0.22) vs. individuals without a cancer history (Δ number of social distancing behaviors = 0.41, 95% CI: 0.31–0.51).

**Table 3:** Associations between metro residency and social distancing measures by cancer history.

	Social Distancing Behaviors^1^		Social Gathering Behaviors^1^		Contacted people outside of household^2^	
	Individual without cancer history LS Mean (95% CI)	Individual with Cancer history LS Mean (95% CI)	Individual without cancer history LS Mean (95% CI)	Individual with Cancer history LS Mean (95% CI)	Individual without cancer history OR (95% CI)	Individual with Cancer history OR (95% CI)
**Age**	0.00 (-0.00, 0.01)	0.02 (0.01, 0.02)	–0.00 (-0.00, 0.00)	–0.01 (-0.01, -0.00)	0.97 (0.96, 0.98)	0.97 (0.96, 0.98)
**Sex**						
Male	ref	–	–	–	–	–
Female	0.08 (-0.02, 0.17)	0.18 (0.10, 0.26)	0.05 (-0.01, 0.12)	0.04 (-0.01, 0.09)	1.25 (1.04, 1.49)	1.13 (0.97, 1.31)
**Race**						
White	ref	–	–	–	–	–
Black/African American	0.27 (0.10, 0.44)	0.62 (0.44, 0.80)	–0.16 (-0.27, -0.05)	–0.29 (-0.40, -0.17)	1.25 (0.89, 1.76)	1.24 (0.87, 1.76)
Asian	0.71 (0.43, 1.00)	0.71 (0.42, 1.01)	–0.42 (-0.60, -0.25)	–0.49 (-0.68, -0.30)	0.678 (0.399, 1.150)	0.507 (0.280, 0.916)
Other/Multiple	0.11 (-0.11, 0.33)	0.30 (0.03, 0.56)	–0.14 (-0.28, -0.00)	–0.14 (-0.31, 0.04)	0.79 (0.52, 1.21)	0.82 (0.49, 1.36)
**Socioeconomic Status group**						
low (score: 0–5)	ref	–	–	–	–	–
intermediate (score: 6–8)	-0.21 (-0.33, -0.09)	-0.08 (-0.18, 0.02)	0.22 (0.14, 0.29)	0.10 (0.04, 0.17)	1.17 (0.93, 1.47)	0.95 (0.78, 1.15)
high (score: 9–10)	-0.20 (-0.32, -0.07)	0.02 (-0.09, 0.13)	0.25 (0.17, 0.33)	0.11 (0.04, 0.18)	0.89 (0.70, 1.12)	0.80 (0.65, 0.98)
**Marital Status**						
Single, never married	ref	–	–	–	–	–
Married/Living as married	-0.12 (-0.27, 0.04)	-0.21 (-0.36, -0.05)	0.01 (-0.09, 0.10)	0.15 (0.04, 0.25)	0.84 (0.62, 1.14)	1.04 (0.77, 1.40)
Divorced/Widowed/Separated/Other	-0.16 (-0.34, 0.02)	-0.23 (-0.40, -0.06)	–0.05 (-0.16, 0.06)	0.08 (-0.03, 0.19)	0.94 (0.67, 1.33)	1.51 (1.09, 2.10)
**# of people in household**	-0.05 (-0.09, -0.02)	0.03 (-0.01, 0.07)	–0.00 (-0.02, 0.02)	–0.01 (-0.04, 0.01)	0.90 (0.84, 0.97)	0.94 (0.87, 1.01)
**Possess device for video conferencing**						
No	ref	–	–	–	–	–
Yes	0.04 (-0.16, 0.24)	0.21 (0.01, 0.40)	0.21 (0.08, 0.33)	0.10 (-0.03, 0.23)	0.97 (0.66, 1.43)	1.06 (0.73, 1.55)
**County of Residence**						
Non-Metro	ref	–	–	–	–	–
Metro	0.41 (0.31, 0.51)	0.13 (0.04, 0.22)	–0.19 (-0.25, -0.13)	–0.12 (-0.18, -0.06)	0.67 (0.55, 0.81)	0.85 (0.72, 1.01)
Interaction: cancer*metro ^3^	P = 0.001		P = 0.12		P = 0.82	

^1^linear regression

^2^logistic regression

^3^Interaction between history of cancer diagnosis and county of residence, included in an overall model for each outcome

When looking at social gathering behaviors, among individuals without a cancer history, those with intermediate and high SES and those who had a video conferencing device reported more social gatherings, whereas individuals who were Black, Asian, and of another race and individuals who lived in metro counties reported fewer social gatherings. Among individuals with a cancer history, those with intermediate and high SES or who were married/living as married reported more social gatherings, whereas those who were older, Black or Asian, and lived in metro counties reported fewer social gatherings. The interaction between cancer history and metro residency was not significant (p interaction = 0.12).

In terms of contacting people outside of the household in the past two weeks, among individuals without cancer, females had a higher odds of contacting people outside of the household (OR = 1.25, 95%CI: 1.04–1.49), whereas those who were older (OR = 0.97, 95% CI: 0.96–0.98), from a household of more people (OR = 0.90, 95%CI: 0.84–0.97), and lived in metro counties (OR = 0.67, 95%CI: 0.55–0.81) had lower odds of contacting any people outside of the household. Among individuals with a cancer history, those who were divorced/widowed/separated had a higher odds of contacting people outside of the household (OR = 1.51, 95%CI: 1.09–2.10), while those who were older (OR = 0.97, 95%CI: 0.96–0.98), Asian (OR = 0.51, 95%CI: 0.28–0.92), and with high SES (OR = 0.80, 95%CI: 0.65–0.98) had lower odds of contacting people outside of the household. The interaction between cancer history and metro residency was not significant.

### Associations between metro residency, social distancing measures, and loneliness by cancer history

After adjusting for age, sex, race, SES, marital status, number of people in household, and possession of a device for video conferencing, individuals who adhered to more social distancing behaviors had higher odds of reporting feeling lonely compared to individuals with lower adherence among both participants with (Table 4, OR = 1.27, 95% CI: 1.17–1.38) and without a cancer history (OR = 1.15, 95% CI: 1.06–1.25). However, only among individuals without a cancer history, it was found that those who contacted a higher number of people outside of the household had lower odds of reporting feeling lonely compared to their counterparts (OR = 0.99, 95% CI: 0.98–1.00).

**Table 4:** Associations between metro residency, social distancing measures, and loneliness by cancer history.

	Individual without cancer history OR (95% CI)	Individuals with cancer history OR (95% CI)
**Age**	0.96 (0.96, 0.97)	0.98 (0.98, 0.99)
**Sex**		
Male	ref	–
Female	1.34 (1.11, 1.60)	1.73 (1.46, 2.04)
**Race**		
White	ref	–
Black/African American	0.80 (0.57, 1.12)	1.72 (0.50, 1.03)
Asian	0.80 (0.47, 1.38)	0.46 (0.23, 0.91)
Other/Multiple	0.80 (0.52, 1.23)	1.09 (0.64, 1.87)
**Socioeconomic Status group**		
0–5	ref	–
6–8	0.88 (0.70, 1.11)	0.75 (0.61, 0.92)
9–10	0.79 (0.62, 1.01)	0.58 (0.46, 0.72)
**Marital Status**		
Single, never married	ref	–
Married/Living as married	0.66 (0.49, 0.89)	0.44 (0.33, 0.60)
Divorced/Widowed/Separated/Other	1.31 (0.93, 1.84)	1.14 (0.82, 1.58)
**# of people in household**	0.89 (0.83, 0.96)	0.99 (0.91, 1.07)
**Possess device for video conferencing**		
No	ref	–
Yes	0.59 (0.40, 0.87)	1.13 (0.75, 1.70)
**Social Distancing Behaviors**	1.15 (1.06, 1.25)	1.27 (1.17, 1.38)
**Social Gathering Behaviors**	0.94 (0.82, 1.06)	0.91 (0.81, 1.02)
**# people contacted outside of household**	0.99 (0.98, 1.00)	1.00 (0.99, 1.00)
**County of Residence**		
Non-Metro	ref	–
Metro	0.97 (0.80, 1.17)	1.15 (0.95, 1.38)
Interaction: cancer*metro	P = 0.01	

The odds of feeling lonely differed by metro residency between individuals with and without a cancer history (p interaction = 0.01). Among individuals without a cancer history, the odds of feeling lonely was lower for those who lived in metro counties (OR = 0.97, 95% CI: 0.80–1.17), whereas among individuals with a cancer history, the odds of feeling lonely was higher for those who lived in metro counties (OR = 1.15, 95% CI: 0.95–1.38). However, these results were not significant.

## Discussion

During the COVID-19 pandemic, the everyday lives of individuals were impacted by the need to social distance [1], which can contribute to feelings of loneliness [3, 4]. Through this study, it was discovered that 69.3% of participants partook in four or five social distancing behaviors. Additionally, 31.5% of individuals did not attend social gatherings and 45.8% were not in close contact with anyone outside of their household. Participants without a cancer history or who had higher adherence to social distancing measures were more likely to report feeling lonely.

While there were no differences in social distancing and social gathering behaviors between participants with and without a cancer history, a smaller percentage of individuals with a history of cancer reported being in close contact with people outside of their household than those without cancer. This latter result supports the hypothesis and prior research finding that cancer survivors were more likely to practice preventive behaviors during COVID-19, such as staying away from crowded areas [5]. Additionally, individuals without cancer may have more outside responsibilities, such as work and school, through which they must contact people outside of their household [21]. While cancer history correlated with differences in the number of social contacts, it was not associated with differences in social distancing and social gathering behaviors. Other factors, such as political affiliation, knowledge about the health risk of COVID-19, and the ability to work from home, may play a larger role in determining these behaviors than cancer history [22, 23].

In addition to examining differences in social distancing practices, our study found that feelings of loneliness varied by cancer history, after adjusting for social distancing behaviors. Contrary to the hypothesis, individuals without cancer were more likely to feel lonely than those with a cancer history. Research examining loneliness in patients with cancer during the COVID-19 pandemic found that this population is susceptible to increased feelings of loneliness, but did not include comparisons to individuals without cancer [8]. However, our finding is supported by a study by van de Poll-Franse, et al. (2020), which discovered that there was a higher prevalence of loneliness in individuals without cancer than those with cancer during the pandemic [24]. This difference could be attributed to how the implementation of social distancing measures was a bigger change for people without cancer than those with cancer, who may be accustomed to decreased social interaction [24]. Additionally, individuals with cancer may have better coping strategies due to their experiences with the disease, allowing them to better adapt to changes from the pandemic [25].

Our study also examined whether the association of social distancing measures and metro residency differed by cancer history. Results found that in both individuals with and without a cancer history, those living in metro countries were more adherent to social distancing behaviors and attended fewer social gatherings. Individuals without cancer living in metro counties also reported contacting fewer individuals outside of their household. This is supported by previous research which found that rural residents were less likely to adhere to COVID-19 preventive behaviors [2, 26]. This could be due to the faster adoption and enforcement of social distancing measures in metro rather than rural areas [7]. Additionally, metro areas tend to have more options for home-delivery services, reducing the need to leave the house [27]. Residents of rural areas may also be less knowledgeable about the threat of COVID-19 and the need to slow its spread [28].

The present study also evaluated how practicing different social distancing measures was associated with feelings of loneliness. Regardless of cancer history, individuals who had higher adherence to social distancing behaviors were more likely to report feeling lonely. This result reinforces previous research finding that increased loneliness was associated with the implementation of social distancing measures during the COVID-19 pandemic [8, 9]. Additionally, it was found that cancer history modified the association between metro residency and social distancing practices. Among individuals without a cancer history, those living in metro counties were less likely to report feeling lonely. This could be explained by how metro residents are more able to stay digitally connected to others, which may alleviate feelings of loneliness [29, 30]. However, among individuals with a cancer history, the odds of feeling lonely was higher in those living in metro counties. The interaction between practicing more social distancing due to being immunocompromised [6] and adhering more to social distancing policies as a metro resident may contribute to higher feelings of loneliness in this population. Further research is needed to examine this interaction.

### Strengths and limitations

Strengths of this study included the large and diverse sample population, which included individuals with and without a history of cancer from different social economic levels in both metro and non-metro counties. Additionally, our study categorized social distancing practices into three scores, allowing us to examine how different behaviors correlated with cancer history and loneliness.

There were several limitations in this study. First, the results relied on the self-reported data from participants, which may have misclassified some responses as the definition of loneliness may vary between individuals. Second, survey responses were collected starting in June of 2020, a couple of months after the enforcement of social distancing policies in the United States. As a result, the findings from this study may differ if the survey had been distributed immediately after social distancing policies were implemented. Third, the cross-sectional nature of this study limited the ability to draw causal relationships. Lastly, the sample was overwhelmingly female and White, which may limit the generalizability of the results to other populations.

Through this study, adherence to social distancing measures during the COVID-19 pandemic among adults living in Ohio and Indiana was evaluated. Additionally, populations susceptible to loneliness during the pandemic, such as individuals without a cancer history or who reported higher adherence to social distancing measures, were identified. As loneliness is a strong predictor of various mental health consequences, such as depression and premature death [31, 32], future studies should examine the prevalence of these mental health outcomes in populations susceptible to feeling lonely during the COVID-19 pandemic. Additionally, further research is needed on how social distancing behaviors and feelings of loneliness varied over time. Lastly, while some interesting interactions between metro residency and other associations were discovered in this study, more research is needed to support these findings.

In conclusion, our findings suggest that individuals without a history of cancer were more likely to report feeling lonely during the COVID-19 pandemic than those with a history of cancer. Additionally, individuals who participated in more social distancing measures were more likely to feel lonely than those who partook in fewer measures. Results from this study and future research can inform efforts to support the mental health of vulnerable populations impacted by the COVID-19 pandemic.

## Supporting information

S1 Table Demographic characteristics between participants included and excluded from the analysis. (PDF)
