# Supplementary material for: Associations between cancer history, social distancing behaviors, and loneliness in adults during the COVID-19 pandemic
Source: PLoS One. 2023 Feb 16;18(2):e0281713. doi: 10.1371/journal.pone.0281713 (PMC9934343; doi:10.1371/journal.pone.0281713)
Supplement: S1 Table — (PDF) [file pone.0281713.s001.pdf]

S1 Table. Demographic Characteristics between Participants included and excluded from the analysis

Variable	Total (n=9280)	Included (n=5729)	Excluded (n=3551)	p-value
**Age, mean±SD, yrs**	56.84 ±13.63	56.69±13.59	57.09±13.69	0.164
**Sex**				
Male	3102 (33.60%)	2039 (35.59%)	1063 (30.35%)	<.0001
Female	6130 (66.40%)	3690 (64.41%)	2440 (69.65%)	
**Race**				
White	8201 (89.46%)	5119 (89.35%)	3082 (89.65%)	0.096
Black/African American	559 (6.10%)	335 (5.85%)	224 (6.52%)	
Asian	162 (1.77%)	112 (1.95%)	50 (1.45%)	
Other/Multiple	245 (2.67%)	163 (2.85%)	82 (2.39%)	
**Marital Status**				
Single, never married	924 (10.02%)	532 (9.29%)	392 (11.22%)	0.0005
Married/Living as married	6771 (73.41%)	4282 (74.74%)	2489 (71.22%)	
Divorced/Widowed/Separated/Other	1529 (16.58%)	915 (15.97%)	614 (17.57%)	
**Socioeconomic Status group**				
low (score: 0-5)	1754 (23.69%)	1252 (21.85%)	502 (29.97%)	<.0001
intermediate (score: 6-8)	3081 (41.61%)	2355 (41.11%)	726 (43.34%)	
high (score: 9-10)	2569 (34.70%)	2122 (37.04%)	447 (26.69%)	
**Total number of people in household**				
1	1482 (16.23%)	911 (15.9%)	571 (16.79%)	<.0001
2	4299 (47.09%)	2609 (45.54%)	1690 (49.69%)	
3	1552 (17.00%)	1026 (17.91%)	526 (5.76%)	
≥4	1797 (19.68%)	1183 (20.65%)	614 (18.05%)	
**Possess device for video conferencing**				
No	559 (6.07%)	262 (4.57%)	297 (8.55%)	<0.0001
Yes	8643 (93.93%)	5467 (95.43%)	3176 (91.45%)	
**County of Residence**				
Non-Metro	3005 (32.64%)	1463 (25.54%)	1542 (44.34%)	<0.0001
Metro	6202 (67.36%)	4266 (74.46%)	1936 (55.66%)	
**Cancer History**				
No	4353 (49.81%)	2583 (45.07%)	1771 (58.92%)	<0.0001
Yes	4387 (50.19%)	3147 (54.93%)	1240 (41.18%)	
